# Dynamic Step‐Down Tasks Reveal Altered Lower Limb Biomechanics in Chronic Ankle Instability

**DOI:** 10.1002/jfa2.70102

**Published:** 2026-01-09

**Authors:** Nader Farahpour, Usef Mohammadi Yaghoubi, Shawn Robbins, Paul Allard, Gabriel Moisan

**Affiliations:** ^1^ Department of Sport Biomechanics, Faculty of Sport Sciences Bu‐Ali Sina University Hamedan Iran; ^2^ Department of Physiotherapy McGill University Montreal Quebec Canada; ^3^ Department of Kinesiology Montreal University Montreal Quebec Canada; ^4^ Department of Human Kinetics Université du Québec à Trois‐Rivières Trois‐Rivières Quebec Canada; ^5^ Groupe de Recherche sur les Affections Neuromusculosquelettiques (GRAN) Trois‐Rivières Quebec Canada

**Keywords:** chronic ankle instability, joint motion, landing, lower limb, muscle moments, step‐down

## Abstract

**Background:**

Accurate knowledge about biomechanical alterations in chronic ankle instability (CAI) during dynamic movements may inform rehabilitation strategies.

**Objective:**

To identify movement patterns associated with CAI injury during a step‐down task.

**Method:**

Seventeen participants with CAI and 17 healthy controls performed a step‐down task from heights of 20 and 40 cm. Lower limb joint angles, range of motion (ROM), moments, and power were measured. The one‐dimensional statistical parametric mapping (SPM) test compared groups across the entire task (0%–100%).

**Results:**

At 20 cm height, the CAI group exhibited greater hip abduction angles (0%–2%, *p* = 0.024 and 21%–77%, *p* = 0.014) but smaller hip abduction (7%–13%, 19%–20%, and 47%–64% (*p* < 0.05)), hip external rotation (8%–12% and *p* = 0.04), knee abduction (7%–30%, *p* = 0.001 and 49%–53%, *p* = 0.024), and ankle external rotation moments (7%–42% and *p* = 0.001). At 40 cm height, the CAI group showed greater hip abduction (44%–100% and *p* = 0.005), reduced ankle eversion (4%–12% and *p* = 0.012) angles, and smaller hip abduction, hip external rotation, knee abduction, ankle plantarflexion, and external rotation moments (all *p* < 0.05). No between‐group differences were observed for the ROMs and power (*p* > 0.05).

**Conclusion:**

CAI individuals exhibited greater hip abduction, less ankle eversion, and smaller muscle moments, which are associated with an increased risk of injury. Rehabilitation should emphasize strengthening the hip muscles to mitigate the risk of injury.

## 1 Introduction

Acute ankle sprains, involving serious stretching or tearing of the ankle ligaments, affect approximately 7 per 1000 people annually in the general population, based on U.S. emergency department data [1]. These injuries account for 15% of all reported injuries among collegiate athletes [2, 3], with lateral ankle sprains representing 70% of cases [2]. Females, children, and individuals participating in indoor or court sports are considered the highest‐risk subgroups [4].

Chronic ankle instability (CAI) often develops following inadequately treated or incompletely rehabilitated lateral ankle sprains [5, 6, 7]. Among active populations, CAI has a prevalence of 25%, with incidence rates ranging from 7% to 53% [8]. A systematic review reported that 46% (range: 9%–76%) of individuals with a history of ankle sprains develop CAI [8].

CAI is characterized by recurrent sprains or episodes of the ankle “giving way,” which is a sensation of sudden instability during weight‐bearing activities, pain, swelling, impaired balance, and diminished postural control [5, 9]. These deficits stem from proprioceptive impairments, including reduced joint position awareness and poor neuromuscular coordination, which may lead to instability during dynamic movements [10]. Consequently, maladaptive movement patterns, such as altered gait, landing strategies, and compensatory mechanisms, may elevate stress on adjacent joints (e.g., knee, hip, lower back), predisposing individuals to further injuries [11, 12, 13, 14]. CAI may lead to the development of post‐traumatic osteoarthritis [9]. Additionally, a fear of reinjury can reduce participation in physical activity, perpetuating a cycle of deconditioning [15, 16].

Understanding the mechanisms underlying CAI is critical for developing effective prevention and rehabilitation strategies to mitigate its long‐term consequences. Previous research on CAI has explored various kinematic and kinetic alterations, such as changes in joint angles, moments, and muscle activation, during functional tasks [17, 18, 19, 20], confirming that these biomechanical deviations are a hallmark of CAI. Reported findings include greater ankle inversion, plantarflexion [20], knee and hip flexion [21], increased knee extension moment, reduced peroneal activation [18], and proprioception deficit [22, 23]. Given that the mechanism of a lateral ankle sprain involves excessive inversion, internal rotation, and plantarflexion, an increase in any of these movements may be considered a risk factor for injury.

However, the literature is marked by inconsistency, with other studies reporting contrasting results, such as less knee flexion and greater ankle dorsiflexion [18, 21, 24]. These discrepancies suggest that proprioceptive deficits and biomechanical alterations in CAI are likely task‐dependent [22].

A significant limitation contributing to this ambiguity is that previous studies have often modeled the foot as a single segment, neglecting to analyze the entire lower limb kinetic chain concurrently. Our study addresses this gap by reporting comprehensive angles, moments, and power for all lower limb joints. Critically, by employing the Rizzoli foot model [25], we measured motion and forces at the hip, knee, ankle, midfoot, and hallux. This multisegment approach is essential for generating a more accurate understanding of the biomechanical alterations associated with CAI.

To investigate these alterations, we focused on a dynamic step‐down task. This challenge is particularly valuable for assessing kinematic and kinetic responses as it simulates daily activities and is central to rehabilitation protocols [22, 26]. Despite its relevance, studies employing this methodology have yielded inconsistent findings, and the effect of a key variable, landing height, has been rarely examined. Analyzing step‐down tasks from varying heights may provide deeper insights into instability mechanisms under different demands, ultimately informing more targeted and effective rehabilitation strategies.

This study expands on existing literature by comparing the angles, moments, and power of the lower‐limb joints between individuals with CAI and healthy controls during step‐down tasks from two heights (20 and 40 cm). By clarifying these biomechanical differences, we aim to enhance understanding of biomechanical alterations in individuals with CAI and assess how step‐down height influences movement patterns. Our findings may inform clinical practice and athletic training protocols. Specifically, we aimed to (1) evaluate the effects of CAI on lower‐limb biomechanics during step‐down tasks and (2) examine the interaction between CAI and step‐down height. We hypothesized that: (a) Individuals with CAI would exhibit altered kinematics and kinetics, including reduced ankle eversion, greater inversion angles, and smaller eversion moments; (b) greater step‐down height would amplify these biomechanical alterations (particularly at the ankle and knee), increasing sprain risk.

## 2 Methods

### 2.1 Participants

In this study, 17 individuals with CAI formed the CAI group and 17 active healthy individuals served as the control group (CG). All participants in both groups were male athletes completing at least three regular exercise sessions per week. The CAI group was recruited from provincial sports federations and orthopedic clinics, whereas the CG was recruited solely from the provincial sports federations, between September 2022 and May 2024. All participants were right‐footed, with limb dominance determined by having them kick a thrown ball. In the CAI group, the injured limb was invariably the dominant (right) foot. Among CAI participants, only the dominant right foot was injured, with the nondominant left foot unaffected.

Demographic characteristics for both groups and clinical measures for the CAI group are summarized in Table 1. Clinical measures for the CAI group included: the Foot and Ankle Ability Measure for Activities in Daily Life (FAAM‐ADL) and Sports (FAAM‐S), the Cumberland Ankle Instability Tool (CAIT), the number of self‐reported ankle sprains sustained, and the frequency of ankle giving way episodes during the past 6 months.

**TABLE 1:** Participant characteristics and CAI clinical measures.

Variables	CG	CAI	Sig.
Age (years)	26.8 ± 3.2	28.5 ± 3.9	0.178
Height (m)	1.79 ± 0.06	1.78 ± 0.05	0.652
Body mass (kg)	82.5 ± 14.0	82.4 ± 12.2	0.98
BMI (kg/m²)	25.3 ± 3.7	25.7 ± 3.0	0.721
Number of ankle sprain	—	6.0 ± 2.9	—
Number of giving ways in 6 months	—	4.8 ± 1.9	—
Time since the first AS (years)	—	5.0 ± 2.6	—
Time since the last AS (years)	—	1.3 ± 0.5	—
FAAM_ADL %	—	80.1 ± 6.8	—
FAAM_S %	—	68.9 ± 8.4	—
CAIT (out of 30)	—	15.6 ± 5.2	—

The inclusion criteria were based on the standards provided in the International Ankle Consortium statement [9]. Participants were included in the CAI group if they (a) had a history of at least one lateral ankle sprain (LAS) occurrence at least 12 months before the start of the study, (b) experienced recurrent LAS and/or episodes of the ankle‐giving way in the past 12 months, and (c) scored 90% or less on the FAAM‐ADL and 80% or less on the FAAM‐S. Individuals were included in the CG if they had a normal lower limb structure and no history of LAS. Potential participants were excluded from the study if they had any history of major surgery, neuromuscular abnormalities, limb length discrepancy, acute injuries within the 3 months preceding the study, or any clinical issue that could affect landing performance. The Ethics Committee of Hamedan University of Medical Sciences approved the research protocol (IR.BASU.REC.1401.034). Before participation, all participants provided signed informed consent forms.

### 2.2 Tools and Procedure

Body segments' motion and ground reaction forces (GRF) data were collected using a Qualisys motion capture system equipped with eight Miqus cameras (200 Hz) (Version: 2020.3, Qualisys AB, Kvarnbergsgatan 2, 41,105 Gothenburg, Sweden; www.qualisys.com) and two Kistler force plates (1000 Hz). Cameras were mounted on tripods at heights ranging from 1 to 2 m around the laboratory to ensure optimal visibility of the markers. A calibration was performed within a cubic space measuring 2 m in height, 2 m in width, and 4 m in length. The force plates were securely fixed to the ground at the center of the calibrated space. Qualisys Track Manager (QTM) software synchronized and monitored both systems.

**FIGURE 1:**
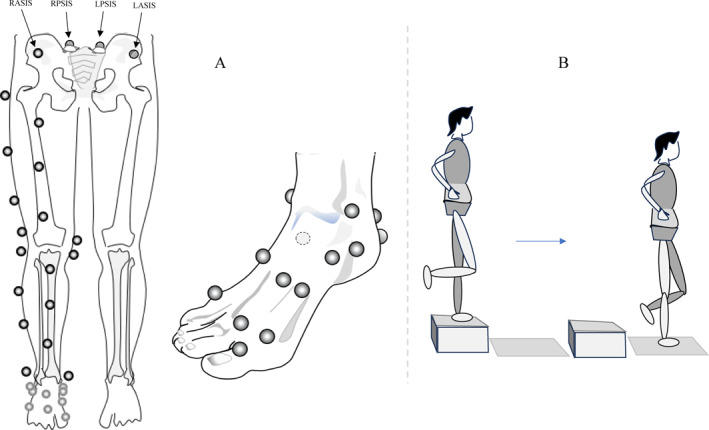
Marker attachment positions are illustrated: (A) Pelvis, thigh, and shank markers based on “full‐body lumbar spine models.” (B) Foot markers adapted from the Rizzoli foot model. (C) Step‐down task setup. LASIS, left anterior superior iliac spine; LPSIS, left posterior superior iliac spine; RASIS, right anterior superior iliac spine; and RPSIS, right posterior superior iliac spine.

Marker placement for the pelvis, thigh, and shank was adapted from the full‐body lumbar spine model [27], whereas the foot marker set was adapted from the Rizzoli foot model [25]. In total, 33 markers were used as follows: 4 markers for the pelvis on the right anterior superior iliac spine (ASIS), left anterior superior iliac spine (LASIS), right posterior superior iliac spine (RPSIS), and left posterior superior iliac spine (LPSIS), and unilaterally on the dominant side which was the injured foot in patients, 8 markers for the thigh, 9 markers for the shank, and 12 markers for the foot (Figure 1A). The foot markers were further divided to segment the foot into three parts: the hindfoot (4 markers), midfoot (7 markers), and hallux (1 marker).

To facilitate the step‐down tasks, two platforms, measuring 20 and 40 cm in height, were positioned 5 cm in front of the force plate.

### 2.3 Tasks

To perform the step‐down task, participants stood on the platform using their nondominant limb, with their dominant foot held off the ground and their hands placed on their waist. They were instructed to step down onto the force plate with their dominant foot (injured foot for the CAI group) and maintain single‐limb balance for 10 s (Figure 1B). Each participant completed four trials for each platform height, totaling eight trials per participant. A one‐minute rest period was provided between trials. Before the main trials, participants engaged in a 5‐min warm‐up session, which included practice step‐downs.

### 2.4 Data Processing

First, the data were digitized using the Qualisys Track Manager (QTM) software and exported as C3D files. A model for each participant was constructed using the Visual3D software, and the angles, moments, and powers of the lower limb joints were calculated using an X‐Y‐Z rotation order. Raw data were filtered before further processing and analysis using a fourth‐order Butterworth low‐pass filter, with cut‐off frequencies set at 6 Hz for kinematic data and 50 Hz for force data. Kinetic variables were normalized to the participant's body weight (%BW). The processed data were exported as text files containing 101 data points, where zero corresponded to the initial contact (defined as GRFZ > 10 N) and 100 corresponded to full knee extension postlanding. Additionally, the range of motion (ROM) for each joint was calculated.

### 2.5 Statistical Analysis

The Shapiro–Wilks test and the “spm1d.stats.normality.ttest2” open‐source test were performed to examine the normality of the distributions in peak values and the time‐series data, respectively. In the SPSS 27 environment, MANOVA and repeated‐measure ANOVA tests were used to compare the between‐group and within‐group differences for the ROM values of all variables. For the time‐series data of each variable, a parametric one‐dimensional statistical parametric mapping (SPM) technique, specifically the independent *t*‐test “spm1d.stats.ttest2,” was employed to compare the patterns between the two groups. For non‐normally distributed data, the nonparametric SPM test “spm1d.stats.nonparam.ttest2” was used. The significance level for all comparisons was set at *p* < 0.05.

## 3 Results

The two groups had no significant difference in age, height, body mass, or BMI.

### 3.1 Angles

The overall range of motion (ROM) across the lower limb joints (Table 2) did not show significant differences between the CAI group and the CG (*p* > 0.05). Significant effects of the joint factor in within‐group comparisons revealed that, during the step‐down task, the ankle, knee, hip, and midfoot displayed the highest to lowest sagittal plane ROM, respectively (*p* < 0.001 for all combinations). This pattern was consistent across both groups.

**TABLE 2:** ROM (°) of the hip, knee, ankle, midfoot, and hallux joints during stepping‐down from 20 to 40 cm heights (mean ± SD).

Joint	Height	Sagittal			Frontal			Transverse		
		C	CAI	Sig	C	CAI	Sig	C	CAI	Sig
Hip	20 cm	12.45 ± 3.96	11.75 ± 3.00	0.568	5.81 ± 2.02	6.42 ± 2.27	0.411	6.03 ± 1.85	5.54 ± 1.84	0.446
	40 cm	19.12 ± 3.50	19.24 ± 4.09	0.928	9.82 ± 2.41	10.20 ± 3.04	0.688	9.96 ± 2.19	9.39 ± 2.72	0.507
	Sig	0.001	0.001		0.001	0.001		0.001	0.001	
Knee	20 cm	22.25 ± 5.06	20.81 ± 6.21	0.466	3.53 ± 1.68	2.97 ± 1.28	0.282	10.03 ± 2.94	10.04 ± 2.18	0.992
	40 cm	33.43 ± 5.34	36.24 ± 5.87	0.154	4.40 ± 2.30	4.60 ± 1.23	0.745	12.55 ± 2.85	12.83 ± 2.92	0.779
	Sig	0.001	0.001		0.09	0.001		0.001	0.002	
Ankle	20 cm	37.61 ± 3.15	38.62 ± 5.74	0.529	5.93 ± 3.16	4.62 ± 2.60	0.198	10.18 ± 3.30	8.99 ± 2.30	0.233
	40 cm	42.85 ± 3.82	44.67 ± 5.53	0.271	6.95 ± 2.81	5.97 ± 2.80	0.315	11.78 ± 3.14	11.08 ± 2.44	0.447
	Sig	0.001	0.001		0.02	0.118		0.001	0.001	
Midfoot	20 cm	10.82 ± 3.26	9.39 ± 2.74	0.175	3.13 ± 1.85	2.74 ± 1.40	0.49	5.09 ± 1.66	4.04 ± 1.40	0.054
	40 cm	13.68 ± 3.14	11.91 ± 3.56	0.135	3.49 ± 1.76	3.28 ± 2.01	0.747	5.82 ± 1.65	5.20 ± 1.79	0.297
	Sig	0.001	0.001		0.273	0.175		0.005	0.001	
Hallux	20 cm	10.59 ± 4.55	9.48 ± 4.49	0.479						
	40 cm	16.42 ± 4.76	14.03 ± 7.63	0.283						
	Sig	0.001	0.001							

Additionally, significant effects of the height factor (*p* < 0.001) indicated that increasing the step‐down height resulted in greater ROM in all joints. A significant interaction between the height and joint factors was demonstrated, showing that increasing the step‐down height from 20 to 40 cm resulted in the largest ROM changes in knee flexion, hip adduction, and hip internal rotation (*p* < 0.05). This height × joint interaction was consistent across both groups.

**FIGURE 2:**
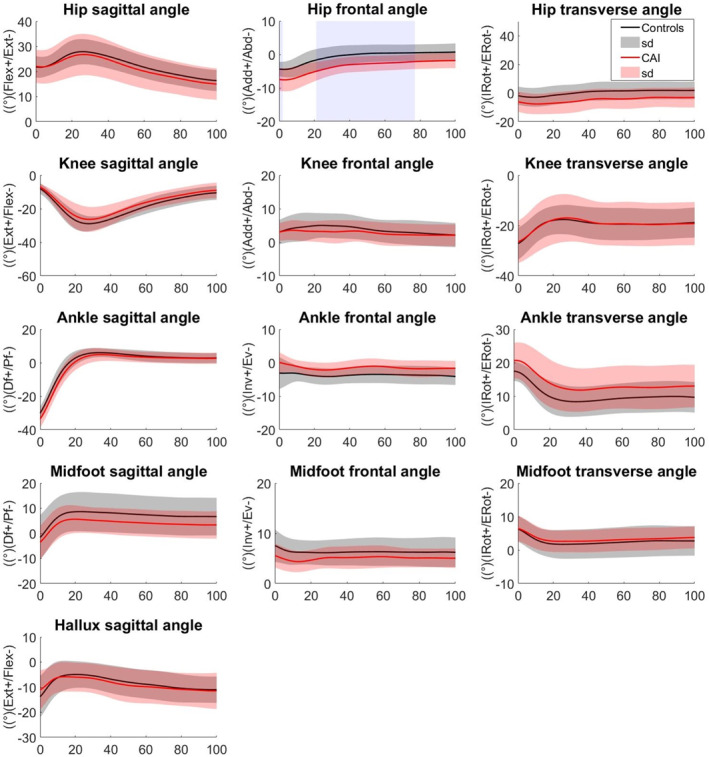
Lower limb joint angles (°) during step‐down from 20 cm height in EG and CG. Significant areas are gray‐shaded.

**FIGURE 3:**
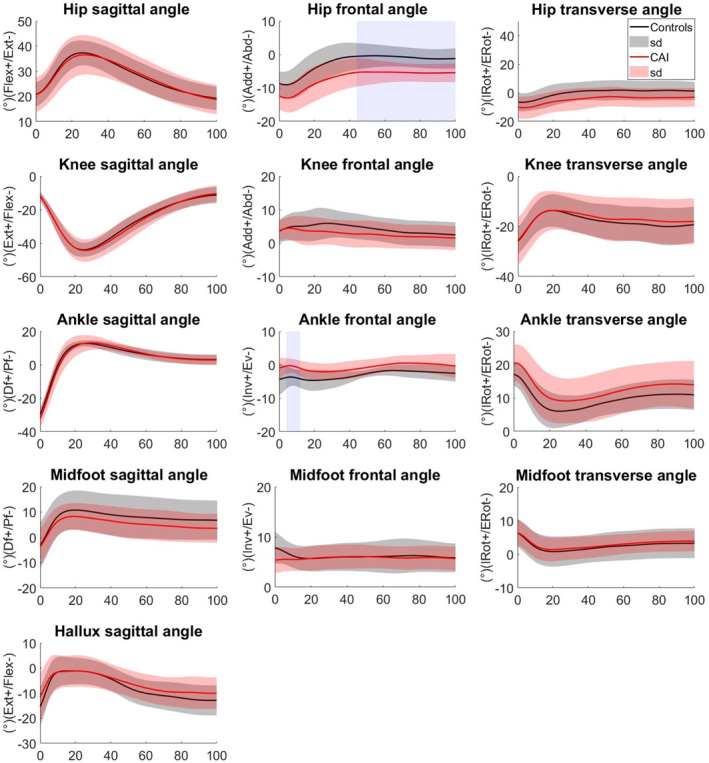
Lower limb joint angles (°) during step‐down from 40 cm height in EG and CG. Significant areas are gray‐shaded.

The SPM analysis revealed that the CAI group exhibited greater hip abduction at 0%–2% (*p* = 0.024) and 21%–77% (*p* = 0.014) in stepping down from the 20 cm platform. They also exhibited greater hip abduction from 44% to 100% (*p* = 0.005) and smaller ankle eversion from 4% to 12% (*p* = 0.012) than the CG in the 40 cm platform condition (Figures 2 and 3).

### 3.2 Moments

The repeated‐measure analysis with significant effects for both “joint” (*p* < 0.001) and “height” (*p* < 0.001) factors revealed that in the sagittal plane, the joints' moments were significantly different from each other, with the ankle, knee, and hip ranked from higher to lower moments (*p* < 0.001). Moments in stepping down from 40 cm height were greater than in stepping down from 20 cm height (*p* < 0.001).

**FIGURE 4:**
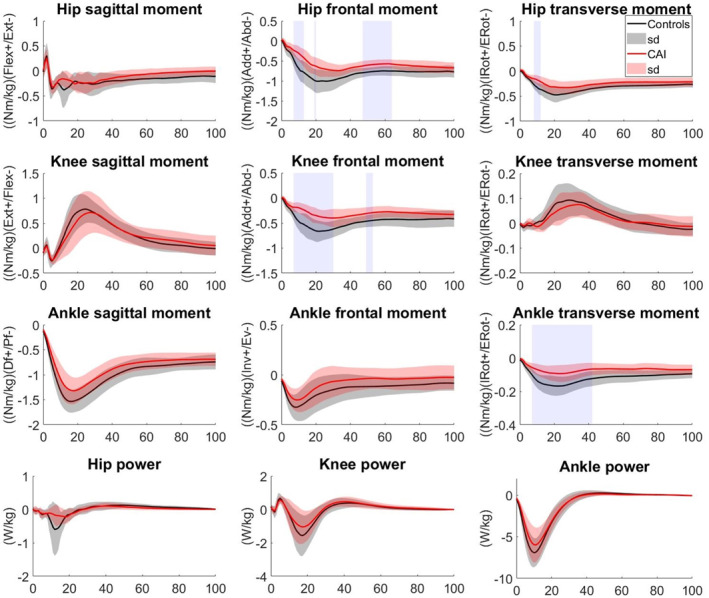
Lower limb joint moments and powers in step‐down from 20 cm height in EG and CG. Significant areas are gray‐shaded.

The SPM between‐group comparisons showed that in stepping down from 20 cm height (Figure 4), the hip abduction moments from 7% to 13% (*p* = 0.023), 19%–20% (*p* = 0.049), and 47%–64% (*p* < 0.001) as well as the hip external rotation moment from 8% to 12% (*p* = 0.040) were smaller in CAI than in CG. Also, the knee abduction moment from 7% to 30% (*p* = 0.001) and 49%–53% (*p* = 0.024), and the ankle external rotation moment from 7% to 42% (*p* < 0.001) of the stepping down from 20 cm height in the CAI group were significantly smaller than in the CG (Figure 4).

**FIGURE 5:**
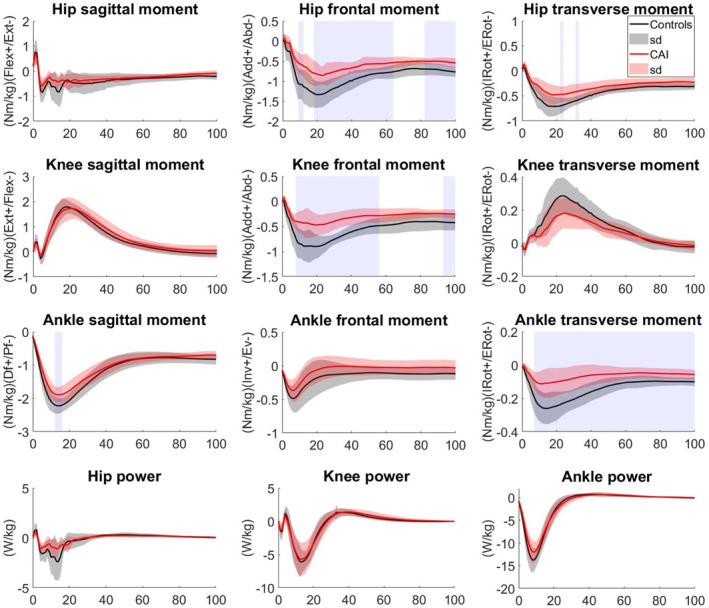
Lower limb joint moments and powers in step‐down from 40 cm height in EG and CG. Significant areas are gray‐shaded.

During the stepping down task from 40 cm height, the hip abduction moment from 9% to 12% (*p* = 0.035), 18%–64% (*p* < 0.001), and 82%–100% (*p* < 0.001), and the hip external rotation moment from 22% to 24% (*p* = 0.041) and 31%–33% (*p* = 0.028) were smaller in CAI group than in CG. The knee abduction moment from 8% to 56% (*p* = 0.001) and 93%–100% (*p* = 0.014) of the step‐down task, as well as in the ankle plantarflexion moment from 12% to 16% (*p* = 0.048), and external rotation moment from 7% to 100% (*p* = 0.001) of the task were smaller in CAI group than in CG (Figure 5).

### 3.3 Power

The repeated‐measure comparisons on peak power also showed significant effects for the “joint” (*p* < 0.001) and “height” (*p* < 0.001) factors. The joint powers ranked from the greatest to smallest were the ankle, knee, and hip. Increasing the height also increased power in all joints (*p* < 0.001). However, the influence of height varied across the joints (*p* < 0.001).

In all joints, negative power was explored, absorbing the impact force of the step‐down. The SPM comparisons revealed that the differences in joints' power between the CAI and CG were nonsignificant throughout the stepping‐down from both heights (Figures 4 and 5).

## 4 Discussion

The present study investigated the angle, moments, and power alterations in the lower limb joints during step‐down tasks from 20 to 40 cm heights in individuals with CAI compared to healthy controls.

Our results revealed that although overall ROM of the lower limb joints remains preserved in individuals with CAI, critical deviations manifest in the dynamic pattern of specific joints' angles and moments. Notably, the CAI group demonstrated greater hip abduction and reduced ankle eversion, reflecting a greater tendency toward ankle inversion during the step‐down task from a 20 cm height, which became more pronounced when landing from a 40 cm height. Stepping down from a higher platform increased knee flexion, hip adduction, and hip internal rotation compared to other joint motions. These findings confirm our first and second hypotheses that CAI alters movement patterns that are clinically associated with the condition, which is amplified with greater step‐down height.

Lateral ankle sprains most frequently occur through mechanisms involving excessive inversion, plantarflexion, and internal rotation during weight acceptance [28, 29]. A moderately everted ankle position during landing is theorized to be a protective neuromuscular strategy as it facilitates dissipation of inversion torque at the talocrural and subtalar joints [23]. Our observation of diminished ankle eversion, reflecting an inversion propensity, in individuals with CAI during step‐down tasks is a movement pattern associated with ankle injury mechanisms. This aligns robustly with prior studies demonstrating greater inversion angles during the load‐bearing phase of landing in CAI cohorts [23, 30, 31]. The attenuated ankle eversion response may stem from peroneal muscles weakness and altered neuromuscular recruitment [32], consistent with Delahunt et al.’s [33] report of delayed peroneus longus activation correlating with precontact inversion. Simpson et al. [23] further observed that CAI individuals exhibit not only larger maximum inversion angles but also shorter time to peak inversion during tilted‐surface landings.

This propensity for ankle inversion in our CAI group underscores a critical failure in sensorimotor adaptation and highlights the imperative for targeted evertor strengthening in rehabilitation paradigms [29].

Contrasting with some studies reporting reduced ankle dorsiflexion in CAI as an injury risk factor [24, 33], our findings revealed no significant sagittal‐plane ankle motion differences, a result concordant with multiple step‐down studies [34, 35, 36]. These discrepancies likely arise from methodological variations across studies, including differences in clinical cohort characteristics, task design and its dynamic or static nature, performing the task with or without footwear, and analytical approaches. Our use of multijoint SPM analysis during dynamic step‐downs reveals that CAI adaptations predominantly manifest in frontal‐plane control and kinetic patterns, deficits potentially masked in isolated‐joint or static assessments.

At the knee, individuals with CAI exhibited no significant alterations in movement in the sagittal, frontal, or transverse planes compared to the control group. This finding aligns with studies reporting comparable knee flexion during controlled step‐down tasks [19, 34, 35]. However, this contrasts sharply with protocols that impose rapid force absorption demands, such as jump landings [36] and stop‐jump maneuvers [24], where reduced knee flexion is consistently documented. This task‐dependent divergence suggests that CAI individuals employ fundamentally different compensatory strategies based on movement context: during reactive high‐velocity activities, a protective stiff‐knee strategy enhances joint stability by reducing degrees of freedom, whereas controlled descents permit proximal redistribution of biomechanical demands.

Crucially, our kinetic analysis revealed that despite preserved sagittal‐plane angles, the CAI group exhibited significantly reduced frontal‐plane knee moments, indicating the pattern of global moment reduction and optimized load dissipation. This emphasizes that task design intrinsically modulates compensatory mechanisms in CAI. This kinetic finding is consistent with the established proximal link between ankle and knee dynamics as highlighted in recent systematic reviews [37, 38]. Specifically, the smaller knee abduction moment suggests the ground reaction force passed closer to the knee joint center, reducing the muscular demand on the primary knee and hip stabilizers to control frontal plane motion. This may represent an attempt to minimize joint loading overall or could point to an underlying deficiency in the neuromuscular control of these stabilizers.

A pivotal finding was the increased hip abduction angle in CAI individuals during step‐down, a result that contrasts with the majority of previous studies, which report greater hip adduction [39, 40] or less hip abduction during landing tasks [41].

In single‐leg standing, the hip abductor of the standing limb is the main postural stabilizer. Lee & Powers [42] demonstrated that diminished hip abductor performance is associated with trunk lean toward the support limb. This adaptation stabilizes the posture by reducing the gravitational moment arm, decreasing the demand from the hip abductors, and could be interpreted as a compensation for the poor hip abductor moment and ankle instability [42].

In contrast to the present result, McCann et al. [41] found less abduction (i.e., more adduction) at the hip joint in patients with CAI during a jump‐landing task. This discrepancy can likely be attributed to the differing task demands; their forward jump‐landing imposes greater reactive forces and requires rapid stabilization after a flight phase, which may necessitate a restrictive strategy of hip adduction to minimize multiplanar instability. In contrast, our controlled step‐down maneuver may permit a different compensatory pattern of excessive hip abduction to offload the ankle. Together, these findings suggest that CAI manifests not as a single movement fault, but as a spectrum of altered strategies, each adopted to meet specific environmental and task demands.

Our findings revealed that individuals with CAI exhibited significantly fewer hip abduction and external rotation moments, fewer knee abduction moments, and fewer ankle plantarflexion and external rotation moments during the step‐down task. Although these kinetic changes may reflect a compensatory strategy to minimize exposure to high‐risk ankle positions, such as excessive plantarflexion, internal rotation, and inversion, they can also indicate the presence of a potential underlying neuromuscular deficit affecting the entire kinetic chain.

The observed reductions in hip abduction and external rotation moments as well as the knee abduction moments, align with the concept of altered movement strategies in CAI, potentially resulting from or leading to weaknesses in key muscle groups essential for dynamic stability. Critically, these findings are aligned well with the work of Lee et al. [43], who demonstrated that diminished hip abductor performance results in medial‐lateral postural instability during step‐down tasks. Our observation of reduced hip abduction moments in CAI individuals, associated with a decreased ankle eversion angle, suggests that a similar proximal instability mechanism may be present, potentially disrupting frontal plane control.

The reduced ankle external rotation moment, combined with increased hip abduction angles observed in the CAI group, may indicate a heightened risk of ankle instability during the step‐down task. Alternatively, these findings could reflect altered joint mechanics aimed at compensating for instability; however, the effectiveness of such an adaptation in preventing further instability remains uncertain.

This proximal instability mechanism likely necessitates compensatory adaptations at distal joints. Lee & Powers [42] provided convincing evidence for the kinetic interactions between the hip and ankle joints. They demonstrated that in healthy individuals, hip abductor fatigue leads to frontal plane postural instability and an increase in peroneus longus activation amplitude, with an earlier onset during landing, which helps stabilize the ankle. Our findings of a significantly reduced ankle external rotation moment, a less pronounced ankle eversion angle reflecting diminished ankle evertor contribution, and reduced control against ankle internal rotation, coupled with greater hip abduction angles, may represent a complex kinetic chain response to chronic ankle instability. The reliance on the greater hip abduction angle, alongside reduced hip abduction and external rotation moments, might be an attempt to passively dissipate reaction forces and offset the loss of functional stability at the ankle, potentially substituting for impaired active control, or indicative of mechanical and/or sensorimotor deficits.

Therefore, two interconnected scenarios may explain the observed global reduction in joint moments in CAI: first, a beneficial compensatory strategy that avoids moments inducing vulnerable ankle positions; second, neuromuscular impairments resulting from hip muscle weakness or diminished motor control, which reduce force generation and coordination throughout the lower limb.

Diminished hip abductor muscle performance, or hip abductor fatigue, can lead to increased center of pressure excursion in the unipedal landing task, reflecting impaired postural control and functional instability at the ankle joint [42, 43]. In such a condition, Lee & Powers [42] showed that the peroneus longus muscle is activated earlier and with greater intensity to enhance lateral ankle stability and compensate for the altered postural demands. In this strategy, the hip joint's dysfunction is compensated for by the ankle adjustment. In our observation, the diminished hip abduction moment might be a compensation for the greater hip abduction angle.

This interpretation reconciles our findings with studies reporting increased ankle plantarflexion moments in CAI during a different task [44], highlighting the task‐dependent nature of these adaptations. The step‐down task, demanding greater frontal plane control, appears to elicit a distinct kinetic strategy emphasizing proximal joint repositioning and global moment reduction, potentially driven by both compensatory intent and underlying proximal deficits impacting stability, as highlighted by [42, 43].

The stepping‐down heights of 20 and 40 cm were chosen to simulate realistic conditions that individuals may encounter in everyday life and athletic endeavors. The differences observed between heights emphasize the effect of increased task difficulty on lower limb biomechanics. Finding reduced contributions from the ankle during descent from the 40 cm height highlights the role of increased dynamic loading and the necessity for greater neuromuscular control, which appears compromised in the CAI group.

The altered angles and moments observed in this study confirm our first and second hypotheses. Increasing the step‐down height was also associated with further alterations in kinematics and kinetics, confirming our second hypothesis. It is plausible that individuals with CAI may face greater challenges during more demanding activities, leading to further risk of injury. Hence, rehabilitation programs should consider task‐specific training that progresses in difficulty to effectively restore lost function and confidence in these individuals. Hopkins et al. [45] found that individuals with CAI, even those with the same inclusion criteria as determined by the International Ankle Consortium [9], may exhibit different movement patterns for the same task, placing them in different vulnerable positions. They suggested clinicians should prepare a treatment plan based on an individual's sensorimotor deficits and movement patterns. These findings underline the need for careful assessment to tailor rehabilitation interventions, and the integration of proprioceptive and neuromuscular training may help correct these maladaptive strategies. Furthermore, the altered mechanics in individuals with CAI can lead to increased stress on other joints and tissues, thus potentially raising the risk of knee, hip, or even lower back injuries. Future research should aim to delineate whether these compensatory strategies have long‐term effects on the kinetic chain beyond the ankle and how they might lead to secondary injury, which may warrant broader clinical attention.

### 4.1 Study Limitations

The present study had a few limitations. First, our sample size was relatively small, although it remained within the minimum acceptable range from a statistical perspective. Additionally, for safety reasons, especially concerning individuals with CAI, we did not include heights greater than 40 cm, which limited our ability to simulate sport‐related movements or more challenging daily living conditions. However, both 20 and 40 cm landing heights are used in most similar studies and are sufficient to show significant differences between the groups. Also, we did not include EMG measurements, which could bring better insight into muscle function. We also failed to recruit female participants in our region. Therefore, these results cannot be generalized for female individuals.

### 4.2 Clinical Implications

Individuals with chronic ankle instability (CAI) exhibit altered lower limb mechanics, including decreased ankle eversion and increased hip abduction angle during landing, which predisposes them to lateral ankle sprains and instability. These changes increase the risk of the ankle “giving way” and may also affect knee alignment, raising injury risks. Although some protective strategies, such as reduced hip and knee abduction moments, are observed during stepping down, they do not fully counteract the poor joint kinematics, including increased hip abduction and a reduced ankle eversion angle. Rehabilitation should improve lower limb joint kinematics and strengthen relevant muscles to address these issues and prevent injury risk.

## 5 Conclusion

This study has demonstrated significant joint angles and moments differences between individuals with chronic ankle instability and healthy controls during step‐down tasks from varying heights. Although the overall range of motion was intact in individuals with CAI, they displayed altered landing mechanics characterized by greater hip abduction and reduced ankle eversion angles as well as decreased hip external rotation and abduction moments, reduced knee abduction, and diminished ankle external rotation and plantarflexion moments. This condition appears to increase the risk of injury. The altered motor control and maladaptive movement patterns are observed in individuals with CAI. The findings advocate for a more individualized approach to rehabilitation, emphasizing strengthening specific muscle groups and incorporating task‐specific training. These findings highlight critical considerations for clinicians' efforts to enhance recovery outcomes and prevent recurrent injuries in individuals with CAI.

## Author Contributions

**Nader Farahpour:** conceptualization, data curation, formal analysis, investigation, methodology, project administration, resources, supervision, writing – original draft preparation, writing – review and editing. **Usef Mohammadi Yaghoubi:** data curation, formal analysis, investigation, writing – review and editing. **Shawn Robbins:** investigation, methodology, writing – review and editing. **Paul Allard:** investigation, writing – review and editing. **Gabriel Moisan:** conceptualization, formal analysis, investigation, methodology, writing – review and editing.

## Funding

The authors have nothing to report.

## Conflicts of Interest

The authors declare no conflicts of interest.

## Data Availability

The data that support the findings of this study are available from the corresponding author upon reasonable request.
